# Generalised machine learning models outperform personalised models for cognitive load classification in real-life settings

**DOI:** 10.3389/fdgth.2025.1650085

**Published:** 2025-10-06

**Authors:** Christoph Anders, Ipsita Bhaduri, Bert Arnrich

**Affiliations:** Digital Health - Connected Healthcare, Hasso Plattner Institute, University of Potsdam, Potsdam, Germany

**Keywords:** human-centered computing, wearable sensors, cognitive load experiments, uncontrolled environment, wavelet decomposition, time-series classification, machine learning, personal assistant

## Abstract

**Introduction:**

By issuing work-break reminders, for example, personal assistants for cognitive load could be beneficial in maintaining health and life satisfaction in society. Wearable sensors facilitate the necessary real-time collection of physiological data. Still, publicly available real-life data sets obtained with wearable sensors are scarce, especially considering multi-modal recordings. Furthermore, data is usually recorded in either completely *controlled* or *uncontrolled* environments, missing the opportunity to study participants across optimal laboratory and realistic real-life settings.

**Methods:**

This work collected data from ten university students during given and self-chosen cognitive load tasks, resembling typical working environments from over 40% of the OECD population, and investigated if commercially available sensors suffice for building cognitive load assistants. The study design accounted for a balanced distribution of eight working hours per participant, split between *controlled* and *uncontrolled* environments.

**Results:**

Across participants, no single feature correlated significantly with cognitive load, but differences in smartwatch indices and biomarkers were identified between low- and high-load scenarios. Generalised machine learning models like Logistic Regression achieved F1 scores of up to 0.91, 0.77, and 0.54 for two, three, and five-class classification, respectively.

**Discussion:**

The presented study design marks a step towards real-life mental state assistants, and the anonymised dataset was made publicly available.

## 1 Introduction

Cognitive load (CL) was defined by (1) as “*the difference between the capacities of the information processing system that are required for task performance to satisfy performance expectations and the capacity available at any given time*”. Three additive aspects of CL have since been distinguished in the field, namely germane (*acquisition of knowledge*), intrinsic (*task-specific*), and extraneous (*information design*) load as outlined by (2). According to (3), stress can be defined as arousal during an uncontrollable challenge leading to anxiety in an individual. Stress and mental workload can be connected factors found to modulate each other, as presented by (4), which can lead to cognitive overload and fatigue (5).

If high levels of CL and stress are prolonged and regularly exceed the individual’s capacities, the individual can develop serious health consequences, ranging from fatigue over depression to cardiovascular diseases (6). One of the United Nations’ sustainable development goals (SDG) is healthy lives and well-being for all at all ages, according to the (7). Therefore, modulators need to be found to accurately identify and intervene in situations of prolonged cognitive overload and stress. The recent surge in wearable devices, as surveyed and quantified by (8), is likely to support this cause. An example of an existing application is continuous heart monitoring through smartwatches. As presented by (9), such applications can issue an alarm via the wearable following a coronary event and subsequently suggest or automatically call for medical care. While these applications consider some immediately life-threatening conditions, events occurring over longer periods remain underaddressed. Smartwatch manufacturers present various (stress) indices to bridge this gap, but the proprietary nature of their algorithms hinders an evaluation of their accuracy in addressing challenges regarding prolonged CL and stress.

Across populations, knowledge workers are especially at risk for cognitive overload and prolonged (mild) stress levels. Setz et al. (10) used wearable electrodermal activity (EDA) sensors to distinguish CL from stress. Muaremi et al. (11) used smartphone activity, questionnaires, and a chest belt to classify the stress levels of 35 participants over four months, without strict control over the activities. Work-interruption management—based on CL classification—was investigated by (12). Following a machine learning challenge on CL classification of data from the Microsoft Band 2, (13) found that the submitted answers struggled to overcome intersubject variability. Wilson et al. (14) investigated an eye-tracker and the Empatica E4 in classifying CL in a plane’s cockpit, finding Photoplethysmogram (PPG) and EDA features to be most indicative. Giorgi et al. (15) found a strong positive correlation between data from wearable sensors—particularly the Empatica E4 and Muse S—and gold-standard versions of the underlying measurement technologies. Fine et al. (16) investigated CL for pre-defined tasks across 21 participants and found indicators of heart-rate variability to be correlated with CL.

Gasparini et al. (17) investigated classifying CL during movement-related (non-office) tasks, and found that task type as well as CL level could be classified using PPG, Electromyography (EMG), and Galvanic Skin Response (GSR) data. It is important to note that, while similar modalities and sensors can be used, CL and (mild) stress classification are distinct from activity recognition. To exemplify, while the movement patterns used to identify a walking activity, the CL might be vastly different if the individual is relaxing or focused on a phone call. Hence, the same activities can be combined with arbitrary levels of CL.

González Ramírez et al. (18) performed a scoping review of 40 studies on stress management and found most experiments were conducted in controlled environments, underlining the need for more real-life experiments. An example of such experiments is the work of (19), which focused on classifying laboratory-task-induced stress using physiological and video data. Liu et al. (20) used an eye-tracker and the Empatica E4 to classify CL levels during cooking activities. Beiramvand et al. (21) utilised mobile electroencephalogram (EEG) and showcased that discrete wavelet transform (DWT) features could be used to distinguish mental workload in an imbalanced three-class classification problem. While (22) investigated CL in both controlled and uncontrolled environments and published over 315 h of wearable sensor data, their work did not consider office tasks comparable across participants. A systematic review of CL found few works on real-life tasks, such as office or learning tasks (23), while practitioners suggest a combination of CL concepts (24), and many data collections use well-established tasks, such as the N-Back contrasted with the operation of semi-autonomous vehicles (25).

**Figure 1:**
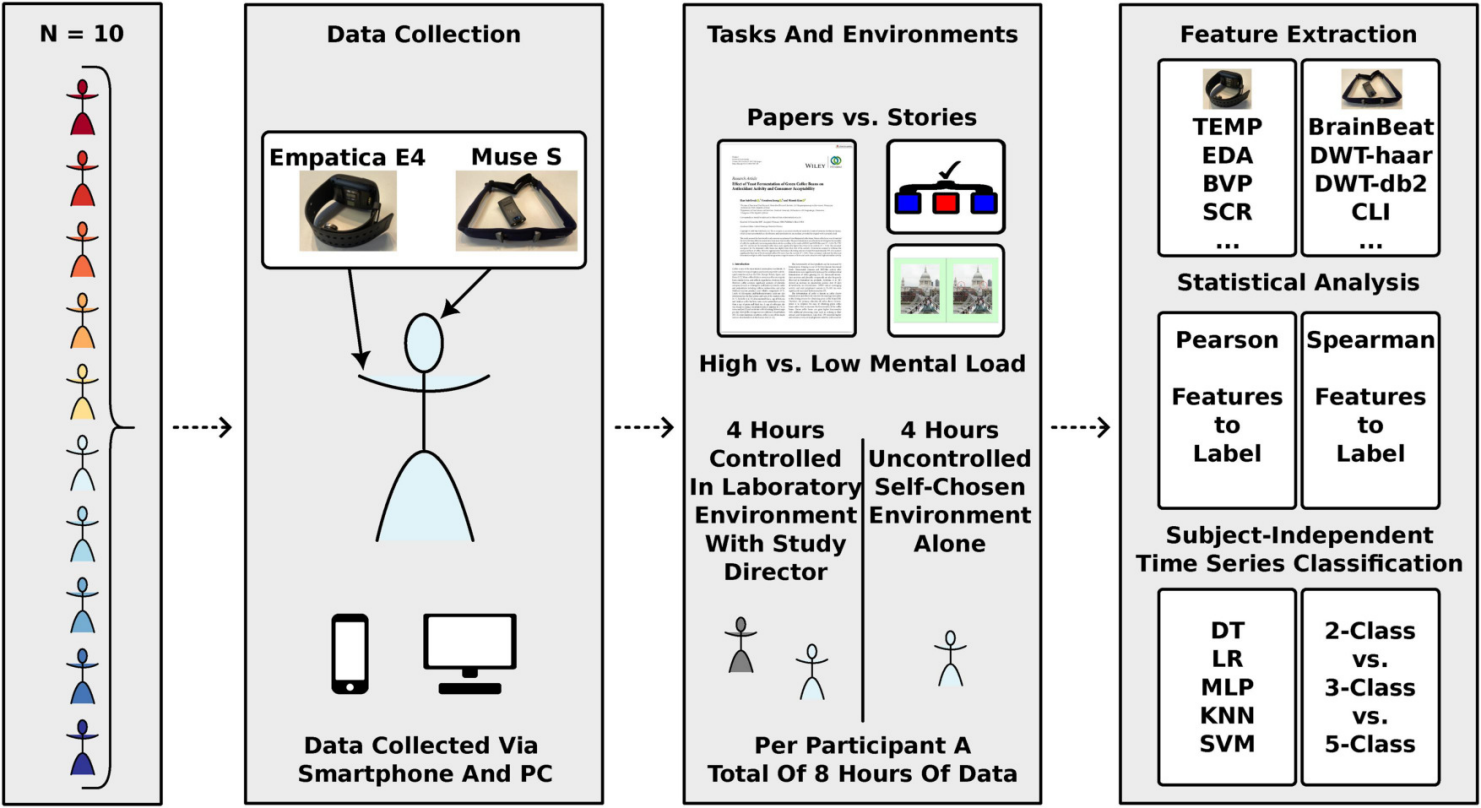
Schematic overview of the study design and data analysis pipeline. Participants enrolled in the experiment were provided with the wearable sensors Empatica E4 and Muse S. Each participant recorded data for four hours in a controlled laboratory environment and four hours in self-chosen environments. Screenshot of scientific publication reproduced from: “Effect of Yeast Fermentation of Green Coffee Beans on Antioxidant Activity and Consumer Acceptability” by Han Sub Kwak, Yoonhwa Jeong and Misook Kim, licensed under CC BY 4.0. Image of St. Paul's Cathedral reproduced with permission from: https://www.spotthedifference.com/.

Despite recent advancements, multiple aspects of CL and stress quantification with wearable sensors remain understudied: (i) a mixed-control environment study design, (ii) utilisation of real-life office tasks, and (iii) the investigation of feature importance remains to be explored further. This work contributed to the field by addressing these challenges through a novel study design presented in Figure 1.

## 2 Materials and methods

In line with previous research [e.g., (15, 22)], the wearable sensors Muse S and Empatica E4 were utilised in this study to investigate CL and (mild) stress levels in knowledge workers (i.e., university students). A study design tackling the lack of mixed-control environment study designs utilising real-life tasks was defined.

Participants (n=10; 23 to 51 years old, mean age of 30 years, STD of 8 years; 5 female: 5 male) were invited to a first data recording session, in a regular office room with whiteboards, windows for natural light, and controlled temperature. The room and hallway leading to it were monitored to ensure no interruptions occurred while data was recorded. During this session, the study design was explained, participants were shown how to wear the sensors, and any open questions were answered before the data recording started. While data was recorded, participants were guided through computerised tasks and questionnaires, implemented in Python 3.8 and using the PsychoPy platform (v2022.2.1).

The tasks posed to participants were the Stroop Color and Word Test (SCWT; four colors (*red, blue, yellow, and green*); less than 1.5 s to answer to stimuli), the N-back continuous performance task (two-back; thirteen different colors (*Blue, Yellow, Burlywood, Green, Cyan, Hotpink, Red, Lightpink, Lightsalmon, Lightseagreen, Lightskyblue, Maroon, Olive*); less than two seconds per iteration), and two reading and summarizing tasks. As literature for the reading and summarizing tasks, six scientific publications (26–31) were chosen as difficult texts, and six short-stories from famous English writers [Edgar Allan Poe (“*The Gift of the Magi*”, “*The Masque of the Red Death*”, “*The Cask of Amontillado*”, and “*The Black Cat*”), Oscar Wilde (“*The Devoted Friend*”), and Charlotte Brontë (“*The Search After Happiness*”)] were chosen as easy texts.

A ten-minute relaxation video (first ten minutes from https://www.youtube.com/watch?v=mLwlGsRhNIU) was presented at the beginning of the computerised experiment to ensure that all participants started the experiment calmly. Before the relaxation video and after the last task was completed, the participants performed a one-minute eye-closing session. In between tasks, the participants answered the questionnaires pairwise NASA Task Load Index (NASA-TLX) and Likert-Scales for Mental Workload and Stress, to report on their subjective mental workload level, experienced stress, etc., for the preceding task/activity. In total, each experiment in the controlled environment lasted approximately two hours.

Following the data recording, participants were instructed on how to perform recordings on their own, and the sensors and chargers were handed out alongside a folder with questionnaires and contact information of the study directors. During their uncontrolled data recordings, participants were free to choose the environment(s) in which they would feel most comfortable and natural. There, participants had to follow the pre-defined study protocol for self-chosen environments: eye-closing baseline of one minute, questionnaires, relaxation task of ten minutes, questionnaires, mental workload task (twenty minutes reading and ten minutes summarising of a text), questionnaires, and finally another eye-closing baseline of one minute.

The same questionnaires used in the laboratory recording were used in the uncontrolled environment. As a relaxation task, the spot-the-difference game was utilized (https://www.spotthedifference.com/). A random subset of scientific publications and short stories was chosen for the mental workload tasks, ensuring no text was read twice. Once participants had finished approximately four hours of data recordings in their self-chosen environment(s) (0 to 4 recordings per participant, mean of 2.3 and STD of 1.1 recordings), another appointment for a second recording in the controlled laboratory environment was made.

In the second recording in the controlled laboratory environment, participants completed a second iteration of the same protocol described for the first session. The study protocol and randomisation processes ensured that the scientific publications and short stories were not repeated. Consequently, each scientific publication and short story was read and summarised at most once by the participant. In total, each participant recorded about eight hours of data over the two controlled and n uncontrolled sessions.

Ethical clearance for the study was obtained from the institutional review board under *review number 02/2023*. Inclusion criteria required participants to be between 18 and 68 years old, fluent in English, and to regularly perform performance-evaluated work. Potential participants were excluded from participation if they were either retired or needed to regularly take medication for any neurological disease (e.g., depression, brain damage, or similar). Additionally, pregnant women, participants with hypertension, and participants who could have been in a dependent relationship with the study directors were excluded from participation.

From the Muse S, the data modalities electroencephalography (EEG; sampled at 256 Hz from AF7, AF8, TP9, TP10, and referenced at FpZ, according to the 10–20 system for electrode placement), gyroscope sampled at 50 Hz, as well as photoplethysmography (PPG; sampled at 64 Hz) were recorded. The Empatica E4 recorded skin temperature at 4 Hz, PPG at 64 Hz, electrodermal activity at 4 Hz, and acceleration data at 32 Hz. Once the data was recorded, the questionnaires were digitised, and the respective labels were extracted for each activity performed by the participants. The data collected was published in anonymised form, publicly available without any restrictions from https://doi.org/10.5281/zenodo.15681262.

Additionally, the Muse S and Empatica E4 data were synchronised based on a shaking protocol. For the recordings in the controlled environments, this data was synchronised with the log data stored by the PsychoPy application. Based on timestamps, the labels were aligned with the respective data. Power line interference was filtered by notch-filtering the recorded data at 50 Hz, but no further specific data cleaning or artefact rejection was performed.

Subsequently, the time series data was split into windows of 60 s, and hand-crafted features were extracted. For Muse S, the individual EEG channels (AF7, AF8, TP9, TP10) were used, and various projections were created: *mean* (across all channels), *prefrontal* (mean across AF7 and AF8), *temporal* (mean across TP9 and TP10) and *asymmetry* (right vs. left cerebral hemisphere). For each of the projected channels, band power was extracted using the Fast Fourier Transform for each of the channels Delta (δ,<4 Hz), Theta (θ, 4 Hz to 8 Hz), Alpha (α, 8 Hz to 12 Hz), Beta (β, 12 Hz to 30 Hz), and Gamma (γ, 30 Hz to 45 Hz). Subsequently, various indices were extracted, as reported in the literature. As such, the *Engagement Index* (32), the *Brain Beat* (33) and the *CLI* (34, 35) were extracted. In addition, inspired by related work (36), discrete wavelet transform (DWT) was performed on the notch-filtered as well as the mean data. For the DWT, the Python package *pywt* (https://github.com/PyWavelets/pywt) was used with the two mother wavelet functions “*db2*” and “*haar*”, and the decomposition level was set to eight. Nine of the resulting coefficients were used as features: *cA8, cD8, cD7, cD6, cD5, cD4, cD3, cD2*, and *cD1*. For each of the coefficients, the features *STD, MEAN, MIN, MAX, Skewness, RelativeWaveletEnergy, Kurtosis*, and *ZeroCrossing* were extracted. Per participant, the features were normalized using z-score normalization.

For Empatica E4, various features were extracted from each modality. The *minimum*, *mean*, and *maximum* values for the time window were extracted from the skin temperature. The *minimum* and *maximum skin conductance response*, the *number of skin conductance response peaks*, and the *minimum*, *mean*, and *maximum level of skin conductance* were extracted for each time window from the electrodermal activity signal. The widely used features of *HRV_MeanNN*, *HRV_SDNN*, *HRV_RMSSD*, *HRV_LF*, *HRV_HF*, *HRV_ratio_LF_HF*, and the *minimum*, *mean*, and *maximum heart rate* were extracted from the photoplethysmography signal. Processing of the Empatica E4 data was facilitated by NeuroKit2, presented by (37).

**Figure 2:**
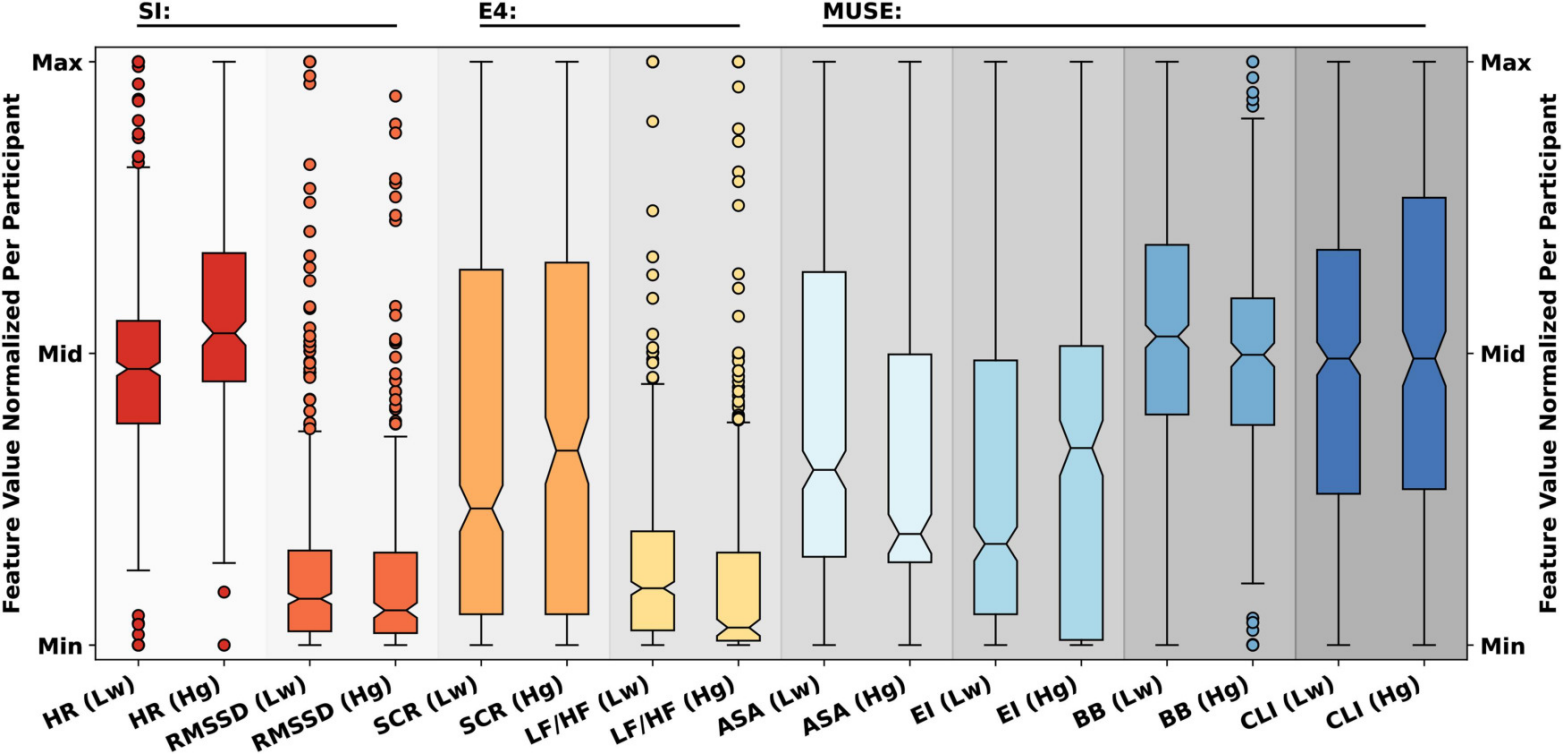
Boxplot comparison of selected features for low (Lw) and high (Hg) cognitive load across participants. Common Smartwatch-Index (SI) features Heart Rate (HR) and Root Mean Square of Successive Differences (RMSSD) used to derive smartwatch stress states [according to (39)]; common E4 features Skin Conductance Response (SCR) and the low Frequency-to-High Frequency (LF/HF) Ratio [according to (40)]; common Muse features ASymmetry index across All channels (ASA), Engagement Index (EI), Brain Beat (BB), and Cognitive Load Index (CLI) [according to (32–35, 41)]. Differences can be seen between HR (SI), LF/HF (E4), ASA and EI (Muse).

From the questionnaires, the mental workload labels were utilized to form three types of classification problems: a two-class classification into low and high mental workload; a three-class classification into low, neither low nor high, and high mental workload; as well as a five-class classification into very low, low, neither low nor high, high, and very high mental workload. As labels, the participant-given labels were extracted from the log files of the PsychoPy experiments as well as from the digitized pen-and-paper questionnaires answered by the participants. An exploratory data analysis of selected features was performed, contrasting low and high load, and the results are visualized in Figure 2.

Based on these mental workload labels, a Pearson correlation analysis and a Spearman correlation analysis were performed to investigate the relationship between the feature values and the labels to analyze how much information a feature provides for mental workload classification, and between the features themselves to analyze which features influence each other and might be redundant. The results from the Spearman correlation analysis were used for a feature selection process to investigate the usage of a smaller subset of features, after it had been found that DWT-features with the “*haar*” mother function consistently provided the best correlation coefficients for binary, three-class, and five-class classification.

The standard machine learning classifiers *DecisionTreeClassifier* (DT), *LogisticRegression* (LR), *MLPClassifier* (MLP), *KNeighborsClassifier* (KNN), and *LinearSVC* (SVM) available in the scikit-learn package (38), were used for time-series classification. In addition to the previously described z-score normalisation, features were min-max normalised. All models were evaluated using leave-one-participant-out cross-validation with both classification accuracy and weighted F1 score as evaluation metrics. The source code for the data processing and the machine learning models was made publicly available via GitHub at https://github.com/HPI-CH/mw_office_2025.

The criteria *gini* and *entropy* were used with the *splitters* best and random and a *max_depth* increasing in 5-point steps from 5 to 305 for the DT classifier. Various combinations of the *solvers* lbfgs, liblinear, sag, and saga were used with the *penalty values* l1, l2, and None for the LR classifier. For the MLP classifier, the *activation function* was chosen to be either logistic, tanh, or relu, and the *hidden layer size* was set to 3, 10, 30, or 50. A *leaf size* ranging from 1 to 50, a *number of neighbors* ranging from 1 to 30, and a *p*-value of either 1 or 2 was used for the KNN classifier. The *penalties* l1 and l2 were used in combination with the *C* values of 0.001, 0.01, 0.1, 1, 10, 100, and 1,000 for the SVM classifier.

## 3 Results

The correlations of the features with the mental workload labels provided by the participants, as quantified with the Spearman Correlation Coefficient, are low. The averaged absolute correlation coefficients rarely exceed a value of 0.08. This strongly suggests that the correlations observed for this general population of ten participants are negligible. For the individuals, however, different features reach low correlation values above 0.2. A similarity across both Pearson correlation analysis and Spearman correlation analysis could be observed: Most of the DWT (*db2*) features (*Kurtosis, ZeroCrossing, MIN, MEAN, and MAX*), and the DWT (*haar*) feature *ZeroCrossing* are present across both sets of features across all electrode positions (*AF7, AF8, TP9, and TP10*) with the highest correlation coefficients with the, by the participants self-assigned, labels for CL, between 0.2 and 0.24.

Due to the large number of automatically extracted features (n=754) using DWT ($n_{DWT-features}=720$), and the insights derived from both the exploratory data analysis and the correlation analysis presented in Figure 2 , the impact of feature selection on time-series classification performance was investigated for three sets of features (*both sensors; Muse S alone; Empatica E4 alone*), as detailed in Table 1.

**Table 1:** For the extracted features, the “x” marks if the feature was included in the reduced feature set, i.e., *Both Modalities Set (20 features)*, *Muse S Set (20 features)*, and *Empatica E4 Set (18 features)*, while the background color indicates from which modality the feature was extracted.

Feature	Both Modalities Set	Muse S Set	Empatica E4 Set
Twelve DWT-features	**x**	**x**	–
TemporalDeltaPower	–	**x**	–
PrefrontalGammaPower	–	**x**	–
TemporalThetaPower	–	**x**	–
TemporalBetaPower	–	**x**	–
TemporalGammaPower	–	**x**	–
EngagementIndex	–	**x**	–
AsymmetryIndexPrefrontal	–	**x**	–
CLI	–	**x**	–
max-scr	**x**	–	**x**
HRV-SDNN	**x**	–	**x**
HRV-RMSSD	**x**	–	**x**
Max-Heart-Rate	**x**	–	**x**
max-skt	**x**	–	**x**
max-scl	**x**	–	**x**
HRV-MeanNN	**x**	–	**x**
HRV-ratio-LF-HF	**x**	–	**x**
min-scr	–	–	**x**
num-scr-peaks	–	–	**x**
min-scl	–	–	**x**
mean-scl	–	–	**x**
min-skt	–	–	**x**
mean-skt	–	–	**x**
HRV-LF	–	–	**x**
HRV-HF	–	–	**x**
Min-Heart-Rate	–	–	**x**
Mean-Heart-Rate	–	–	**x**

The twelve DWT features encompassed mostly DWT detail coefficients cD1, cD7, and cD8 (*i.e., low- and high-frequency information*), furthermore summarized by Kurtosis, Mean, and ZeroCrossing, the specifics of which can be found in the source code made publicly available at https://github.com/HPI-CH/mw_office_2025.

**Figure 3:**
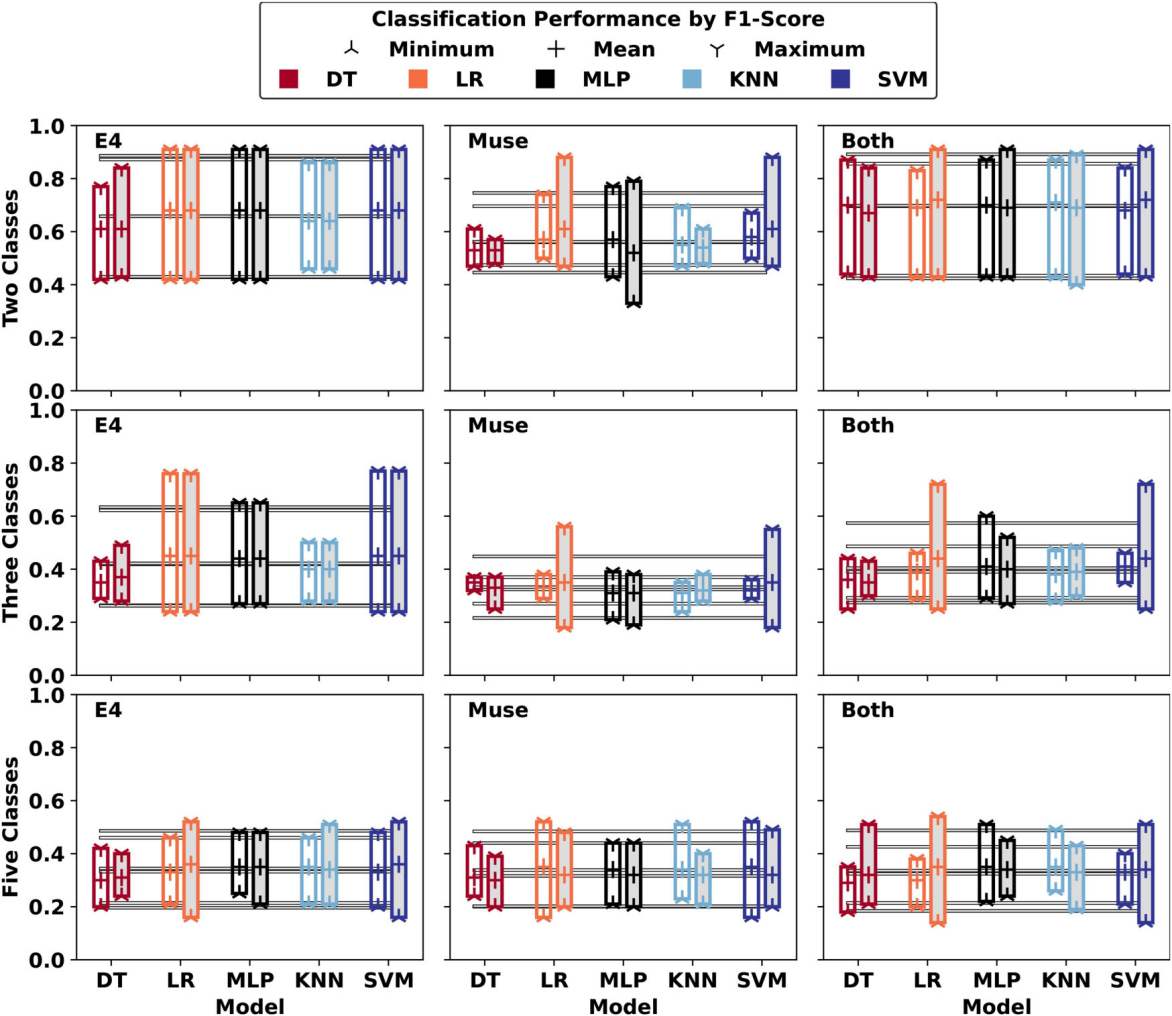
Subject-Independent (Minimum, Mean, Maximum) LOO-CV Results (F1) of generalised models across participant-specific personalised labels. The models which were trained with a reduced feature set selected after feature selection are marked with gray background colour. Average values for (Minimum, Mean, Maximum) across models are given.

The time-series classification performance of generalised Machine Learning models obtained via Leave-One-Participant-Out cross-validation (LOO-CV) is given in Figure 3. The classification performance using all features is given with a transparent background, and the performance of time-series classification using the reduced feature sets is given with a grey background colour. The models DT, LR, MLP, KNN, and SVM show strongly varying classification performances dependent on the classification difficulty. As can be seen across two, three, and five-class classification, the impact of feature reduction on this particular dataset is, however, mostly negligible. While in some cases beneficial, the opposite also holds, and the overall average minimum, mean, and maximum F1-scores of generalised LOO-CV models are nearly identical across classification tasks. For these generalised models, the minimum and maximum F1 scores achieved are 0.33 and 0.91, 0.18 and 0.77, and 0.14 and 0.54 for two, three, and five-class classifications, respectively.

Across the 900 best participant-independent models, most used the following parameters: “*criterion: gini, max_depth:5, splitter: random* (DT)”; “*penalty: l2, solver: lbfgs* (LR)”; “*activation: relu, hidden_layer_sizes: 30* (MLP)”; “*leaf_size: 1, n_neighbors: 9, p: 1* (KNN)”; and “*C: 0.01, penalty: l2* (SVM)”.

**Figure 4:**
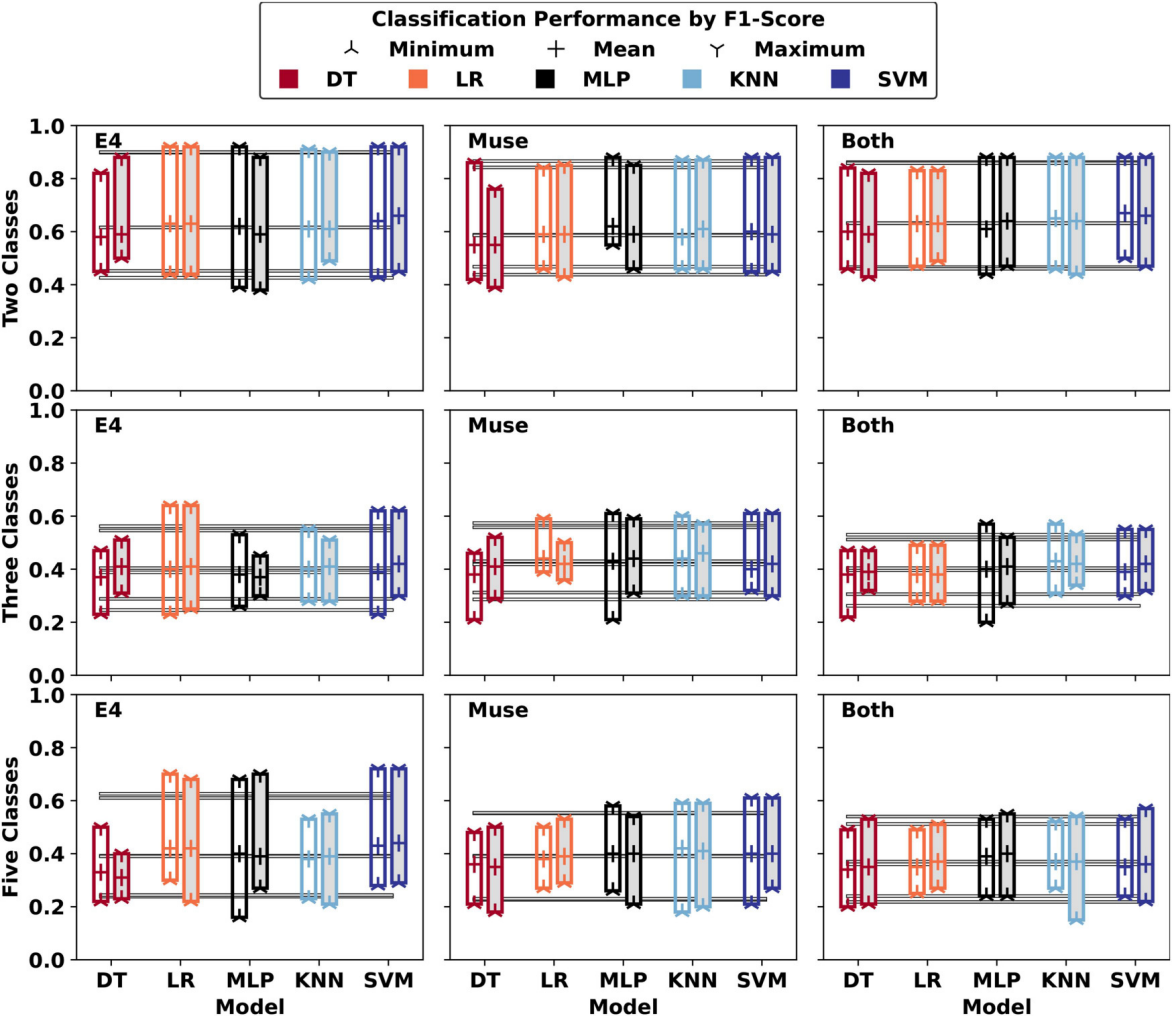
Averaged (Minimum, Mean, Maximum) Five-Fold CV Results (F1) across Participant-Specific Personalised Models. The models that received the cognitive load activity as features are marked with a grey background colour. Average values for (Minimum, Mean, Maximum) across models are given.

Across the literature, the observation can be made that similar wearable devices can be used for activity recognition as well as for CL classification, as exemplified for instance by (42) in their work on human activity recognition (HAR) of activities of daily living, who used the Empatica E4, a device used as well in this work. Hypothesising that context information could be beneficial for CL classification, the idea was investigated how CL classification might change if HAR were able to classify the works done in this experiment. Following through on this thought-experiment, personalised machine learning (ML) models were built using the previously stated reduced feature sets and the activity features *Relaxation, Load, Summary, Reading*, and *Game*, represented as additional one-hot encoded features. The personalised ML models were trained using five-fold cross-validation, and time-series classification results, reported as F1 score, are given in Figure 4. For personalised models, the minimum and maximum F1 scores achieved are 0.38 and 0.92, 0.21 and 0.64, and 0.15 and 0.72 for two-, three-, and five-class classifications, respectively. For this data, the information added by activity features did not significantly change classification results.

## 4 Discussion

While Wavelet Transform is usually used to denoise an input signal as performed by (43), some works also utilize Wavelet Transform to extract meaningful features for subsequent time-series classification [e.g., (44, 45)]. Inspired by this line of investigation, this work utilized Discrete Wavelet Transform (DWT) for feature extraction. In this work, the wavelet-based features extracted from the EEG data were the most stable characteristics in the investigated population. Furthermore, supporting previous research that found the prefrontal cortex to be heavily involved in the processing of mental workload and stress (46, 47), the electrode locations AF7 and AF8 appear ≈1.4 times more often in the most-important features than TP9 and TP10, which are electrode positions usually associated with the encoding of memory and emotions (48).

Building on the underlying idea that context information is important—and that the wearables Muse S and Empatica E4, or the E4’s successor EmbracePlus, could be concealed to enable unobtrusive, real-time CL monitoring—the hypothesis arose that time series classification results might be even better when combining the classification of CL and stress levels with predictions of the type of office activity performed by the individual. In the broader field of mental state classification, (49) pushed conditionally automated driving applications using physiological data by extracting reliable features, yet their work was unable to classify the specific type of task, a necessary step to include context information. However, as presented by (17), it is possible to simultaneously classify the type of task and mental state of a participant using devices similar to those used in this work. If, in the future, a perfect classification of task type would be possible using the same wearable sensors, this information could be used as context information in mental state classification.

This hypothesis was tested in this work for binary, three-class, and five-class classification with reduced feature sets, by passing a numeric value representative of the task type to the ML model as a one-hot encoded feature. The best classification results of generalised models changed to F1 scores (min, avg, max) of [0.43, 0.72, 0.91], [0.24, 0.45, 0.74], and [0.18, 0.34, 0.56], respectively, suggesting at best a minor performance increase (*detailed results not shown*).

For personalised models, a similar negligible change in classification performance was obtained. To summarise, in the evaluation performed in this work, no significant added benefit through additional information about task types could be observed. Future work should investigate how context information could be incorporated differently, and if variability in labels obtained from participants explains current results.

However, this work has two limitations due to the participant and feature selection process applied. Firstly, with n=10, a selection bias might be present regarding the individuals who took part in the study. Secondly, as the feature selection was not performed on a separate part of the data, but rather built on the insights derived from the statistical analysis using all the data, the risk of overfitting is present in the reported results.

Also, future work should focus on combining research on office-work break interface design with mental state classification, as investigated by (50). Consequently, tangible interventions for office work well-being as reviewed by (51), building on targeted and personalized mental workload and stress classification models using physiological data from wearables, would push the workforce of the 21st century closer to the United Nations sustainable development goal (SGD) of “*healthy lives and well-being for all at all ages*”.

This work presented a novel study paradigm enabling researchers to investigate CL and stress in realistic settings with ground-truth values obtained in controlled laboratory environments. Feature importances were investigated, and (assumed) smartwatch indices (SI) were compared with features widely used in the literature. Differences between low and high CL can be seen, especially between HR (SI), LF/HF (Empatica E4), ASA and EI (Muse S). Despite at best low correlation coefficients between extracted features and subjective CL labels, well-performing generalised CL models can be built using standard ML models.

## Data Availability

The datasets presented in this study can be found in online repositories. The names of the repository/repositories and accession number(s) can be found in the article/Supplementary Material.

## References

[1] Gopher D Donchin E. Workload: an examination of the concept. In: *Handbook of Perception and Human Performance, Vol. 2: Cognitive Processes and Performance*. John Wiley & Sons (1986). p. 1–49.

[2] Sweller J. Element interactivity and intrinsic, extraneous, and germane cognitive load. Educ Psychol Rev. (2010) 22:123–38. 10.1007/s10648-010-9128-5

[3] Fink G. Stress, definitions, mechanisms, and effects outlined: lessons from anxiety. In: Fink G, editor. *Stress: Concepts, Cognition, Emotion, and Behavior, Volume 1 of the Handbook of Stress Series*. San Diego: Elsevier inc. (2016). p. 3–11.

[4] Sandi C. Stress and cognition. Wiley Interdiscip Rev Cogn Sci. (2013) 4:245–61. 10.1002/wcs.1222 26304203

[5] Cezar BGS Maçada ACG. Cognitive overload, anxiety, cognitive fatigue, avoidance behavior and data literacy in big data environments. Inf Process Manag. (2023) 60:103482. 10.1016/j.ipm.2023.103482

[6] Kocielnik R Sidorova N Maggi FM Ouwerkerk M Westerink JHDM. Smart technologies for long-term stress monitoring at work. In: *Proceedings of the 26th IEEE International Symposium on Computer-Based Medical Systems (CBMS)*. IEEE (2013). p. 53–8. 10.1109/CBMS.2013.6627764

[7] United Nations Department of Economic and Social Affairs. The sustainable development goals report. In: Ross J, editor. *The Sustainable Development Goals Report 2023: Special Edition*. New York, NY: United Nations (2023). p. 80. 10.18356/9789210024914

[8] Ometov A Shubina V Klus L Skibińska J Saafi S Pascacio P, et al. A survey on wearable technology: history, state-of-the-art and current challenges. Comput Netw. (2021) 193:108074. 10.1016/j.comnet.2021.108074

[9] Guo Y Wang H Zhang H Chen Y Lip GYH. Population-based screening or targeted screening based on initial clinical risk assessment for atrial fibrillation: a report from the Huawei heart study. J Clin Med. (2020) 9:1493. 10.3390/jcm9051493 32429241 PMC7291296

[10] Setz C Arnrich B Schumm J La Marca R Tröster G Ehlert U. Discriminating stress from cognitive load using a wearable EDA device. IEEE Trans Inf Technol Biomed. (2010) 14:410–7. 10.1109/TITB.2009.2036164 19906598

[11] Muaremi A Arnrich B Tröster G. Towards measuring stress with smartphones and wearable devices during workday and sleep. BioNanoScience. (2013) 3:172–83. 10.1007/s12668-013-0089-2 25530929 PMC4269214

[12] Schaule F Johanssen JO Bruegge B Loftness V. Employing consumer wearables to detect office workers’ cognitive load for interruption management. Proc ACM Interact Mob Wearable Ubiquitous Technol. (2018) 2:1–20. 10.1145/3191764

[13] Gjoreski M Mahesh B Kolenik T Uwe-Garbas J Seuss D Gjoreski H, et al. Cognitive load monitoring with wearables–lessons learned from a machine learning challenge. IEEE Access. (2021) 9:103325–36. 10.1109/ACCESS.2021.3093216

[14] Wilson JC Nair S Scielzo S Larson EC. Objective measures of cognitive load using deep multi-modal learning: a use-case in aviation. Proc ACM Interact Mob Wearable Ubiquitous Technol. (2021) 5:1–35. 10.1145/3448111

[15] Giorgi A Ronca V Vozzi A Sciaraffa N Di Florio A Tamborra L, et al. Wearable technologies for mental workload, stress, and emotional state assessment during working-like tasks: a comparison with laboratory technologies. Sensors. (2021) 21:2332. 10.3390/s21072332 33810613 PMC8036989

[16] Fine MS Lombardo JM Colombe JB Gawron VJ Brokaw EB. Use of wearable physiological sensors to predict cognitive workload in a visuospatial learning task. Technol Health Care. (2022) 30:647–60. 10.3233/THC-213106 34397440

[17] Gasparini F Grossi A Giltri M Nishinari K Bandini S. Behavior and task classification using wearable sensor data: a study across different ages. Sensors (Basel). (2023) 23:3225. 10.3390/s23063225 36991935 PMC10055934

[18] González Ramírez ML García Vázquez JP Rodríguez MD Padilla-López LA Galindo-Aldana GM Cuevas-González D. Wearables for stress management: a scoping review. Healthcare. (2023) 11:2369. 10.3390/healthcare11172369 37685403 PMC10486660

[19] Rescio G Manni A Caroppo A Ciccarelli M Papetti A Leone A. Ambient and wearable system for workers’ stress evaluation. Comput Ind. (2023) 148:103905. 10.1016/j.compind.2023.103905

[20] Liu Y Grimaldi NS Basnet N Wozniak D Chen E Zahabi M, et al. Classifying cognitive workload using machine learning techniques and non-intrusive wearable devices. In: *2024 IEEE 4th International Conference on Human-Machine Systems*. Toronto, ON: IEEE (2024). p. 1–6. 10.1109/ICHMS59971.2024.10555690

[21] Beiramvand M Shahbakhti M Karttunen N Koivula R Turunen J Lipping T. Assessment of mental workload using a transformer network and two prefrontal EEG channels: an unparameterized approach. IEEE Trans Instrum Meas. (2024) 73:1–10. 10.1109/TIM.2024.3395312

[22] Anders C Moontaha S Real S Arnrich B. Unobtrusive measurement of cognitive load and physiological signals in uncontrolled environments. Sci Data. (2024) 11:1000. 10.1038/s41597-024-03738-7 39271693 PMC11399273

[23] Seitz J Maedche A. Biosignal-based recognition of cognitive load: a systematic review of public datasets and classifiers. In: Davis FD, Riedl R, vom Brocke J, Léger P-M, Randolph AB, Müller-Putz GR,editors. Information Systems and Neuroscience. Cham: Springer International Publishing (2022). p. 35–52. 10.1007/978-3-031-13064-9-4

[24] Liu X Zhang Y. Re-examining cognitive load measures in real-world learning: Evidence from both subjective and neurophysiological data. Br J Educ Psychol. (2025) 95:446–63. 10.1111/bjep.12729 39696805

[25] Oppelt MP Foltyn A Deuschel J Lang NR Holzer N Eskofier BM, et al. ADABase: a multimodal dataset for cognitive load estimation. Sensors. (2023) 23:340. 10.3390/s23010340 PMC9823940 36616939

[26] Anagnos T Kiremidjian AS. A review of earthquake occurrence models for seismic hazard analysis. Probab Eng Mech. (1988) 3:3–11. 10.1016/0266-8920(88)90002-1

[27] Nunes-Halldorson VS Duran NL. Bioluminescent bacteria: lux genes as environmental biosensors. Braz J Microbiol. (2003) 34:91–6. 10.1590/S1517-83822003000200001

[28] Mansouri S Merhi Y Winnik FM Tabrizian M. Investigation of layer-by-layer assembly of polyelectrolytes on fully functional human red blood cells in suspension for attenuated immune response. Biomacromolecules. (2011) 12:585–92. 10.1021/bm101200c 21306170

[29] Kwak HS Jeong Y Kim M. Effect of yeast fermentation of green coffee beans on antioxidant activity and consumer acceptability. J Food Qual. (2018) 2018:5967130. 10.1155/2018/5967130

[30] Zhao M Li T Alsheikh MA Tian Y Zhao H Torralba A, et al. Through-wall human pose estimation using radio signals. In *2018 IEEE/CVF Conference on Computer Vision and Pattern Recognition*. Salt Lake City, UT: IEEE (2018). p. 7356–65. 10.1109/CVPR.2018.00768

[31] Fernbach PM Light N Scott SE Inbar Y Rozin P. Extreme opponents of genetically modified foods know the least but think they know the most. Nat Hum Behav. (2019) 3:251–6. 10.1038/s41562-018-0520-3 30953007

[32] Apicella A Arpaia P Frosolone M Improta G Moccaldi N Pollastro A. EEG-based measurement system for monitoring student engagement in learning 4.0. Sci Rep. (2022) 12:5857. 10.1038/s41598-022-09578-y 35393470 PMC8987513

[33] Holm A Lukander K Korpela J Sallinen M Müller KMI. Estimating brain load from the EEG. Scientific World J. (2009) 9:639–51. 10.1100/tsw.2009.83 PMC5823228 19618092

[34] Negi S Mitra R. EEG metrics to determine cognitive load and affective states: a pilot study. In *Proceedings of the 2018 {ACM} International Joint Conference and 2018 International Symposium on Pervasive and Ubiquitous Computing and Wearable Computers*. New York, NY: Association for Computing Machinery (2018). p. 182–5. 10.1145/3267305.3267618

[35] Bayrambas F Sendurur E. The comparison of two incidental learning scenarios on a digital learning platform from the cognitive load perspective. Educ Inf Technol. (2023) 29:11087–117. 10.1007/s10639-023-12241-2

[36] Rajaguru H Priyanka GS Anuradha T. Analyzing haar and dB2 with compensatory GMM classifier for epilepsy detection. In: *2022 Smart Technologies, Communication and Robotics*. Sathyamangalam: IEEE (2022). p. 1–4. 10.1109/STCR55312.2022.10009556

[37] Makowski D Pham T Lau ZJ Brammer JC Lespinasse F Pham H, et al. NeuroKit2: A python toolbox for neurophysiological signal processing. Behav Res Methods. (2021) 53:1689–96. 10.3758/s13428-020-01516-y 33528817

[38] Pedregosa F Varoquaux G Gramfort A Michel V Thirion B Grisel O, et al. Scikit-learn: machine learning in python. J Mach Learn Res. (2011) 12:2825–30. Available online at: https://dl.acm.org/doi/10.5555/1953048.2078195

[39] Rosenbach H Itzkovith A Gidron Y Schonberg T. Data from: Assessing Garmin’s stress level score against heart rate variability measurements (2025). 10.1101/2025.01.06.630177 PMC12647429 41292097

[40] Wang H Jiang N Pan T Si H Li Y Zou W. Cognitive load identification of pilots based on physiological-psychological characteristics in complex environments. J Adv Transp. (2020) 2020:5640784. 10.1155/2020/5640784

[41] Cannard C Wahbeh H Delorme A. Electroencephalography correlates of well-being using a low-cost wearable system. Front Hum Neurosci. (2021) 15:745135. 10.3389/fnhum.2021.745135 35002651 PMC8740323

[42] Climent-Pérez P Muñoz-Antón AM Poli A Spinsante S Florez-Revuelta F. Dataset of acceleration signals recorded while performing activities of daily living. Data Brief. (2022) 41:107896. 10.1016/j.dib.2022.107896 35198677 PMC8842007

[43] Joseph G Joseph A Titus G Thomas RM Jose D. Photoplethysmogram (PPG) signal analysis and wavelet de-noising. In: *2014 Annual International Conference on Emerging Research Areas: Magnetics, Machines and Drives*. Kanjirappally: IEEE (2014). p. 1–5. 10.1109/AICERA.2014.6908199

[44] Amin HU Malik AS Ahmad RF Badruddin N Kamel N Hussain M, et al. Feature extraction and classification for EEG signals using wavelet transform and machine learning techniques. Aust Phys Eng Sci Med. (2015) 38:139–49. 10.1007/s13246-015-0333-x 25649845

[45] Merino-Monge M Castro-García JA Lebrato-Vázquez C Gómez-González IM Molina-Cantero AJ. Heartbeat detector from ECG and PPG signals based on wavelet transform and upper envelopes. Phys Eng Sci Med. (2023) 46:597–608. 10.1007/s13246-023-01235-6 36877361 PMC10209290

[46] Kane MJ Engle RW. The role of prefrontal cortex in working-memory capacity, executive attention, and general fluid intelligence: an individual-differences perspective. Psychon Bull Rev. (2002) 9:637–71. 10.3758/BF03196323 12613671

[47] Hänsel A von Känel R. The ventro-medial prefrontal cortex: a major link between the autonomic nervous system, regulation of emotion, and stress reactivity? Biopsychosoc Med. (2008) 2:21. 10.1186/1751-0759-2-21 18986513 PMC2590602

[48] Sauseng P Griesmayr B Freunberger R Klimesch W. Control mechanisms in working memory: a possible function of EEG theta oscillations. Neurosci Biobehav Rev. (2010) 34:1015–22. 10.1016/j.neubiorev.2009.12.006 20006645

[49] Meteier Q De Salis E Capallera M Widmer M Angelini L Abou Khaled O, et al. Relevant physiological indicators for assessing workload in conditionally automated driving, through three-class classification and regression. Front Comput Sci. (2022) 3:1–22. 10.3389/fcomp.2021.775282

[50] Hasan MT Zaman S Wesley A Tsiamyrtzis P Pavlidis I. Sympathetic activation in deadlines of deskbound research—a study in the wild. In: Schmidt A, Väänänen K, Goyal T, Kristensson PO, Peters A, editors. *Extended Abstracts of the 2023 (CHI) Conference on Human Factors in Computing Systems*. New York, NY: Association for Computing Machinery (2023). p. 1–8. 10.1145/3544549.3585585

[51] Brombacher H Houben S Vos S. Tangible interventions for office work well-being: approaches, classification, and design considerations. Behav Inf Technol. (2024) 43:2151–75. 10.1080/0144929X.2023.2241561

